# Evaluation of radiation dose and image quality following changes to tube potential (kVp) in conventional paediatric chest radiography

**DOI:** 10.2349/biij.2.3.e35

**Published:** 2006-07-01

**Authors:** S Ramanaidu, RB Sta Maria, KH Ng, J George, G Kumar

**Affiliations:** 1 Department of Biomedical Imaging, University of Malaya Medical Centre, Kuala Lumpur, Malaysia; 2 Department of Diagnostic Imaging, Paediatric Institute, Kuala Lumpur Hospital, Kuala Lumpur, Malaysia

**Keywords:** paediatric, ESAK, image quality, chest x-ray

## Abstract

**Purpose:**

A study of radiation dose and image quality following changes to the tube potential (kVp) in paediatric chest radiography.

**Materials and Method:**

A total of 109 patients ranging from 1 month to 15 years were included in two phases of the study. Phase 1 investigated the range of entrance surface air kerma (ESAK) values received from patients exposed to the existing exposure factors. In the second phase, new exposure factors using recommended values of tube potential (kVp) with reduced mAs were used. ESAK values were measured using thermoluminescent dosemeters (TLDs). Image quality in both phases was evaluated using image quality criteria proposed by the Council of the European Communities (CEC). Results of both techniques were analysed for any differences.

**Results:**

The overall mean ESAK before the changes was 0.22 mGy (range: 0.05-0.43) Following changes in tube potential, the overall mean reduced to 0.15 mGy (range: 0.03-0.38), a significant reduction by 34%. The interquartile range was reduced from 45% to 40%. However, doses to those below a year in age still remained high. Assessment of image quality was found to have no significant differences as far as the two techniques used were concerned. However, higher image scores were achieved using higher kVps.

**Conclusion:**

Significant dose reduction was achieved through appropriate changes in tube potential and reduction of mAs without any loss in image quality.

## INTRODUCTION

In recent years concern has been raised over the hazards of exposure to small doses of ionising radiation. The probability of a fatal cancer being induced in an individual patient from a single x-ray examination, although small, is dependent on the age of the patient and the type of examination. Exposure during childhood results in a likely two- to three-fold increase in lifetime risk for certain detrimental cancers compared with adults [1]. It is important that the radiation dose to children arising from diagnostic medical exposure is minimised.

Despite rapid development in medical imaging, including the advent of computed radiography and digital imaging, conventional chest radiography remains the most frequent radiological examination among children in major Malaysian hospitals. There are no studies in Malaysia to evaluate the range of radiation dose received by paediatric patients while existing studies on patient radiation doses are mainly done on adult patients [2].

The Commission of European Communities (CEC) has recognised the need for the special treatment of children and has published guidelines suggesting examples of good radiographic practice and present useful image criteria with the aim of producing high quality images at the lowest possible dose to the patient [3]. Good radiographic technique includes the use of optimum kVp. Lower kilovoltages should be avoided in paediatric chest examinations. The CEC recommends the use of 60 - 80 kVp for children between 0-15 years of age. Reduction in patient doses can be achieved through changes in tube potential and the advantage lies in absence of cost implications [4]. Using a higher tube potential has shown a 16-36% reduction in entrance surface doses in neo-natal radiography without any impairment of the diagnostic image quality [5].

The objective of the present study is to identify the level of radiation dose and image quality during paediatric chest radiography. Comparison in dose and image quality will be made following changes to the tube potential being used. Thus far, no studies on the assessment of radiation doses to paediatric patients exist in Malaysia. The present study will provide a useful baseline data to determine doses to paediatric patients. This will allow imaging departments in Malaysia to compare their performances and to undertake necessary remedial actions so that radiation doses to children are minimal.

## METHOD

### General survey of chest radiographic technique

A general questionnaire was distributed to 7 major hospitals in the country to identify the current exposure factors being used in chest radiography of paediatric patients. Superintendent radiographers were required to record the range of kilovoltages (kVp), milliamperes-second (mAs), time and film-screen combination currently used in their respective departments.

### Phases of the study

As the objective of this study was to compare the radiation dose and image quality following changes in tube potential during paediatric chest radiography, the study was conducted in 2 phases. Phase One of the study included survey of radiation dose and an assessment of image quality of the current technique (Technique A) using the existing exposure factors in the radiographic examination of the postero-anterior (PA) and antero-posterior (AP) chest of children. In the second phase, the study was repeated using the recommended technique (Technique B) with tube potential in the range of 60-80 kVp [3]. Both set of results were compared for any differences.

In both phases of the study, radiographic technique was carried out without the use of a secondary radiation grid, consistent with established guidelines on the non-usage of grids during paediatric chest radiography [6]. Exposures were also made without the use of automatic exposure control as the x-ray machine used in this study did not have this facility.

The patients participating in both phases of this study were randomly chosen from the group of patients attending a major Paediatric Radiology Department in Kuala Lumpur, Malaysia. Approximately 18,000 radiographic examinations are performed every year. 51% of these examinations are chest. The department has two x-ray units but only one unit is exclusively used for chest examinations. Exposures were carried out with a 3 phase, 12 pulse generator (Phillips CP 50, Holland) and a Phillips x-ray tube with 17° anode angle. The tube has a total filtration of 2.7 mm Aluminium equivalent. No attempt was made to include any additional filtration.

Variation in kilovoltage was within ± 5 kV while the variation in tube output did not exceed ± 3%. The tube output was found to vary linearly with the timer setting to within 3%. Lanex regular intensifying screens and Agfa-gevaert films were used with Kodak cassettes giving a nominal speed class of 400. Films were processed using a Kodak M4 automatic processor.

### Phase one of the study

Data was collected of patients aged between 1 month and 15 years undergoing AP/PA chest radiography. The initial set of exposure factors, especially selection of kV and mAs or time, was determined by the radiographer using the usual practice of selection of these factors suitable for the patient. The radiographic technique employed depended on the co-operation of the patient. Children who were co-operative and able to stand were done in the erect PA position otherwise the AP supine position was chosen. An FFD of 180 cm was used for erect patients while a FFD of 100 cm was used for supine patients. No attempt was made to identify any differences in dose between these two projections. The x-ray tube was centred to the 4th thoracic vertebrae and collimated to reduce the irradiated area to the minimum. The antero-posterior (AP) thickness of the chest was measured using callipers at the level of the sternal angle.

At the time of the examination patient data: sex, age, height, weight and AP thickness were noted. Exposure parameters recorded were tube voltage (kVp), tube current (mA), time or mAs and FFD. According to the recommendations from the CEC [3], results are represented for separate age groups. Group 0-1 year includes children between one month and 1 year. A child is 1 year old until its 2nd birthday etc. The other groups were 1-5 years, 6-10 years and 11-15 years. Grouping of patients into the recommended age classifications, unfortunately, reduced the number of patients within each sub-group. Comparison of doses were carried out giving due consideration to the variations that might occur due to the small sample sizes. However, the results are expected to indicate general trends in doses hence creating greater awareness of the doses received by paediatric patients.

### Patient dosimetry

Entrance surface air kerma (ESAK) measurements were made by attaching a sachet containing 3 thermoluminiscent dosemeters (TLDs) to the patients’ skin on the central axis of the x-ray beam. The lithium fluoride TLD chips (TL 100, Harshaw) were later read using a TLD reader (Harshaw QS 3500). The TLD system used in this survey was calibrated by the National Radiation Laboratory, New Zealand and found to be performing within recommended levels of precision and accuracy. The calibration of the TLDs was traceable to the National Protocol recommended by the NRPB [7]. The overall uncertainty at the 95% confidence level was ± 20%. The ESAK for each patient was calculated by averaging the readings from the three TLDs in each sachet.

### Assessment of image quality

A checklist based on a modified version of the CEC image quality criteria [3] formed the basis of image quality assessment in the present study, as shown in Table 1. Two radiologists blinded to the study were invited to evaluate the radiographs. This review centred on the visibility of specific anatomical structures. Each viewer was asked to assess the visibility of the structures on a graded scoring system in the range of 1 to 4 from the criterion being cannot be assessed to the structure can be visualised very well. In this scoring scheme the score for each observer would be in the range of 1 to 4 for each criterion and an overall score of 4 to 16 for each radiograph. Higher scores indicate better image quality. The summation of all the statements in the questionnaire led to the assessment of radiographic quality of the image and represented features that were dependent on the radiographic technique used for the examination, particularly the tube voltage.

**Table 1:** Criteria used to evaluate image quality

**Criteria**	**Visualisation of :**
A	Vascular pattern in central two-third of the lungs
B	Trachea and the proximal bronchi
C	Diaphragm and costo-pherinic angles
D	Retrocardiac lung and mediastinum

### Phase two of the study

Once approval was obtained from the senior radiologist of the department to introduce the “recommended technique” (Technique B), which included raising the tube voltage following that recommended by the CEC, a new set of exposure factors were then derived, with the minimum set at 60 kVp for all age range. Choice of kVp was based on the antero-posterior thickness. Results of the AP thickness in the first phase showed the minimum thickness was approximately 8 cm which formed the lower limit of the scale. Each increase in 1 cm followed an increase of 2 kV.

Increase in kVp necessitates a decrease in mAs to maintain similar image density. Reduction in mAs was made using the common radiography practice of an increase of 15% in kVp requires a decrease of 50% in the mAs value [8]. The radiographer selects the appropriate kVp after measuring the AP thickness. The appropriate reduction in mAs was then calculated and the new exposure was made. To maintain the consistency of the film density in both phases of the study, readings of the optical density at selected anatomical positions were recorded and compared.

Radiation dosimetry, image quality assessment, recording of patient data and exposure factors were similar to those carried out in the first phase. Radiographs of patients in the second phase were evaluated using the same protocols and by the same two radiologists in the first phase of the study.

### Statistical analysis

Data obtained in the survey was analysed using the SPSS statistical package. Large samples (n>30) which were normally distributed were analysed using parametric statistical tests (e.g. t-test, Pearsons r). Sub-groups with smaller numbers were analysed using non-parametric tests (e.g. Mann-Whitney, Kruskal-Wallis) [9].

## RESULTS

### Radiographic technique in major hospitals

**Figure 1:**
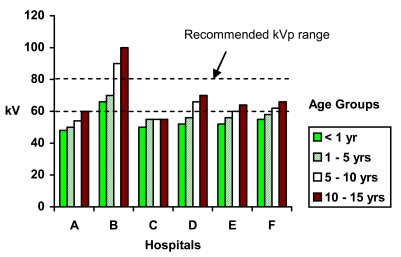
Range of Tube Potential Used By Various Hospitals During Paediatric Chest Radiography.

The preliminary survey to identify the range of kVps used in seven major hospitals showed most of the centres were using tube potentials below 60 kVp during paediatric chest radiography, particularly in children below 5 years old (Figure 1). Only one centre was using tube potentials above 60 kVp. Due to time constraints no ESAK measurements were done in this initial survey. Evaluation of other parameters, e.g. type of equipment, tube filtration, film-screen combinations and exposure time were excluded due to insufficient respondent information.

### Patient Data

A total of 109 patients between the ages of 1 month to 15 years were included in the study. Table 2 summaries the patient parameters accrued in the survey. 43 patients were examined in the first phase of the study using the existing exposure factors, while 66 patients were examined following the use of new exposure factors, i.e. with the change in tube potential following the values recommended by the CEC. The overall mean age was 4.5 years (range: 1 month to 12 years).

**Table 2:** Patient data [mean ± standard deviation (range)] from both techniques

**Age Groups**	**Sample size**	**Age** **(years)**	**Weight** **(kg)**	**Height** **(cm)**	**A-P Thickness** **(cm)**
**0 - 1 year**					
Technique A	15	0.6 ± 0.3 (0.08 - 1.0)	6.9 ± 1.7 (3.5 - 9.3)	68.3 ± 5.1 (53.0 - 85.0)	11.8 ± 1.3 (9.0 - 13.0)
Technique B	19	0.7 ± 0.3 (0.08 - 1.0)	7.2 ± 1.7 (3.5 - 10.5)	69.0 ± 8.5 (55.0 - 90.0)	11.4 ± 1.1 (9.0 - 13.0)
**1 - 5 years**					
Technique A	13	2.6 ± 0.7 (1.5 - 4.0)	12.2 ± 2.5 (8.5 -17.3)	92.0± 8.5 (81.0 - 108.0)	13.5± 1.4 (12.0 - 17.0)
Technique B	19	2.6 ± 1.2 (1.5 - 4.0)	12.3 ± 3.3 (7.8 - 20.0)	91.4 ± 10.0 (80.0 - 112.0)	12.5 ±. 0.94 (11.0 -15.0)
**6 - 10 years**					
Technique A	9	7.6 ± 1.3 (6.0 - 9.0)	18.7 ± 8.0 (9.0 - 36.0)	117.7 ± 20.0 (78.0 - 145.0)	14.1± 1.2 (12.0 - 16.0)
Technique B	18	7.0 ± 1.4 (6.0 - 9.0)	19.2 ± 4.8 (13.4- 35.0)	117.6 ± 11.2 (101.0 - 143.0)	13.8 ±. 1.0 (12.0 -16.0)
**11 - 15 years**					
Technique A	6	10.8 ± 1.7 (10.5 - 12.0)	25.6 ± 5.9 (17.8 – 35.5)	135.8 ± 5.9 (128.0 - 141.0)	15.7± 1.8 (13.0 - 18.0)
Technique B	10	11.3 ± 1.0 (11.5 - 12.0)	32.2 ± 5.8 (25.0 - 39.0)	142.0 ± 9.7 (126.0 – 160.0)	15.5 ± 1.8 (12.0 -19.0)

### Applied tube potential and ESAK

A summary of the applied tube potential, mAs and ESAK values obtained before and after the change in applied tube potential during both phases (techniques) is shown in Table 3.

**Table 3:** Comparison of applied kVp, mAs and Entrance Surface Air Kerma (ESAK) between Technique A and Technique B

	**Technique A**			**Technique B**		
**Age Group**	**kVp**	**mAs**	**ESAK**	**kVp**	**mAs**	**ESAK**
**< 1 yr**						
Sample size	15	15	15	19	19	19
Mean	47.9	4.7	0.27	61.2	1.81	0.21
S.D	5.4	0.8	0.05	1.30	0.30	0.08
Min.	45	1.7	0.18	60	1.10	0.04
Max.	62	5	0.36	64	2.20	0.34
**1 - 5 yrs**						
Sample size	13	13	13	19	19	19
Mean	57.5	4.5	0.22	65.2	2.0	0.15
S.D	8.08	1.1	0.09	5.7	0.17	0.06
Min.	46	2	0.11	60	1.6	0.07
Max.	70	5	0.37	74	2.3	0.27
**5 - 10 yrs**						
Sample size	9	9	9	18	18	18
Mean	63.9	4.1	0.18	73.1	1.9	0.09
S.D	7.4	1.6	0.11	2.5	0.22	0.04
Min.	47	2.0	0.05	62	1.5	0.03
Max.	72	6.0	0.39	70	2.3	0.17
**10 - 15 yrs**						
Sample size	6	6	6	10	10	10
Mean	63.5	5.2	0.17	76.6	2.2	0.10
S.D	3.3	0.57	0.05	3.3	0.19	0.02
Min.	58	5.0	0.08	73	2.0	0.06
Max.	68	6.4	0.21	83	2.5	0.13

The existing range of kVps used in the first phase of the study (Technique A) showed kVps higher than 70 were used in older children (above 10 years) while lower kVps below 60 kVp were predominant among younger children aged below 5 years. In the second phase of the study, the minimum kVp was set at 60 kVp. The mean applied kVp for the all the groups increased from 56.3 to 67.8, an increase of 20%, which was statistically significant (t-test, p<0.05).

### Effect of change of tube potential on entrance surface dose (ESAK)

The mean ESAK before the changes for the all the patients was 0.22 mGy (range 0.05-0.39). Following the increases in kVp the mean ESAK was 0.15 mGy (range 0.03-0.34) which was significantly lower that the ESAK obtained during Technique A (t-test, p<0.05). Overall reduction of 34% in the ESAK was achieved following an increase of 20 % in kVp and a reduction of 57% in mAs values

**Figure 2:**
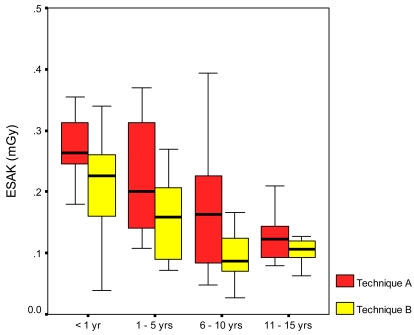
Range of Entrance Surface Air Kerma (ESAK) within age sub-groups from both techniques.

Variations in ESAK within subgroups are shown in Figure 2 in the form of a box-whisker plot. There was a significant reduction of doses received by the 0-1 year old group during Technique B. Observations of the doses received in Technique B showed a trend in lower doses in all the sub-groups. The highest reduction was achieved in the 5-10 years age group i.e. an increase of 14% in kVp produced a reduction of 50% in ESAK.

**Figure 3:**
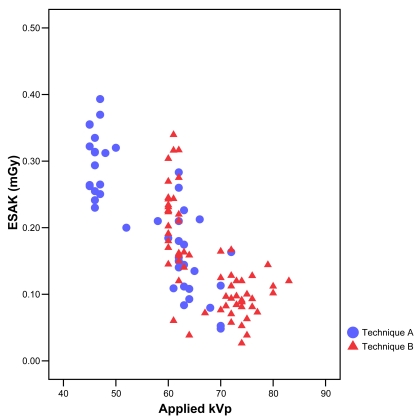
Relationship between Entrance Surface Air Kerma (ESAK) and applied tube potential.

Plotting the values of ESAK against kVp for both techniques produced a scatter gram as shown in Figure 3. The variation of ESAK with applied kVp displayed the trend towards lower ESAKs with increased kVp.

**Figure 4:**
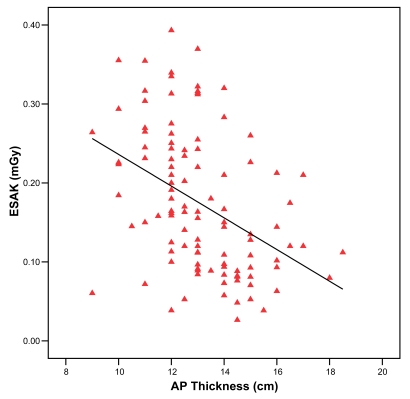
Relationship between Entrance Surface Air Kerma (ESAK) and AP thickness.

The exposure given to a particular patient, hence the dose received, is closely related to the antero-posterior (AP) thickness of the patient’s chest. Figure 4 shows the relationship between ESAK and the AP thickness of the patients. A negative correlation was seen indicating lower doses were achieved with increasing body thickness.

### Effect of change in kVp on image quality

#### Image Criteria Score

From the 109 patients a total of 48 radiographs were retrieved for image evaluation and analysis. 24 radiographs were acquired from the first phase and the second phase of the study. The poor retrieval rate was due to problems of tracing the films back from the wards and clinics as at the time of the study the filing system in the Paediatric Institute was being reorganised.

**Figure 5:**
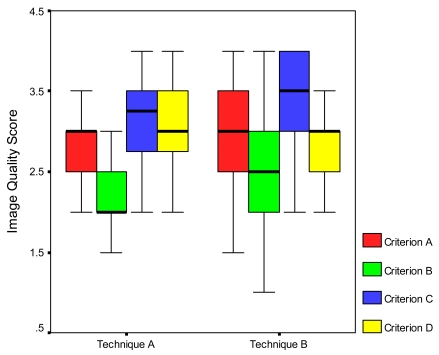
Comparison of image scores for each criterion.

Image quality of the chest radiographs was assessed in order to investigate if the radiographs produced with higher kVp were of inferior quality. The overall average radiologists’ scores for both sets of radiographs from the two techniques showed no significant statistical differences although the overall scores of the images obtained during Technique B were slightly higher. Analysis of the individual scores for each criterion showed some variations as shown in Figure 5. There was an increase in values for individual criteria scores within Technique B except for the visualisation of the retro-cardiac lung and mediastinum (Criterion D). Wide variations were noted in the visualisation of Criterion B (trachea and the proximal bronchi).

### Optical density measurements on selected areas on all radiographs.

Film density on selected areas on all radiographs was measured to compare the influence of altering kVp on the image density. A summary of the densities measured are shown in Table 4. While the results indicate slightly higher densities recorded under the new technique, there were no significant differences between both sets of density measurements.

**Table 4:** Comparison of optical density measurements between Technique A and Technique B

	**Technique A**			**Technique B**		
**Area**	**Mean**	**S.D**	**Range**	**Mean**	**S.D**	**Range**
Mediastinum	0.31	0.06	0.23 - 0.48	0.34	0.09	0.20 - 0.49
Left lung	1.48	0.56	0.76 - 2.33	1.56	0.45	0.66 - 2.42
Right lung	1.40	0.54	0.69 - 2.19	1.41	0.45	0.68 - 2.22

## DISCUSSION

This survey indicated that there was a common practice of selecting lower kVps during paediatric chest radiography in major Malaysia hospitals. This finding is not surprising since many radiographers and radiologists believe that the small size of children particularly the younger age group, necessitates use of lower kVp to enhance radiographic contrast. The range of kVps used in these hospitals was similar to the range used in the present study before recommending any change in tube potential.

The overall range of kVps used for all age groups in the first phase of the study was between 45-76 kVp. Following changes the range increased to between 60-82, a mean increase of 20%. There was substantial decrease in the mAs (52%), a major contributor to patient dose. Tube potential below 60 kVp, the lower bound of the level recommended by CEC was predominant for children below 5 years of age, where there were occasions of kVps below 50 kVp. In the second phase of the study, the mean increase for the below 1 year and 11-15 years age group increased by 28% and 21% respectively. The middle two groups had smaller increases of 14% as the existing exposure factors were already in the above 60 kVp range hence it did not necessitate major increases.

The overall mean ESAK before the changes was 0.22 (range 0.05-0.43). Higher doses were predominant among the younger age group while lower ESAKs were found in older children. The magnitude of the spread is of concern since the examinations were done in one room over a period of few weeks. This is indication of the variations of patient size and preference of individual radiographers for selection of different exposure doses. Doses seemed to decrease with age and the doses were much higher in children below 1 year. Doses were much higher that the achievable ESAK of 0.07 mGy recommended by the CEC for a 10 month old child [3]. The slightly higher doses in the younger children could be due to the common practice among radiographers to use lower kVps and higher mAs to enhance radiographic contrast. Following changes in the tube potential in the second phase of the study, the overall mean ESAK was 0.15 mGy, a significant reduction of 34%. The interquartile range was reduced from 40% to 45% indicating that the spread of doses had been greatly reduced. However, there still existed a wider range of doses particularly in the under 1-year-olds.

The prevalence of higher doses among younger children could also be due to the influence of technical parameters, namely additional filtration. The total tube filtration of the x-ray tube in the present study was 2.7 mm aluminium equivalent without the inclusion of any additional filtration. The CEC recommends the use of additional filtration of up to 1mm aluminium plus 0.1-0.2 mm copper. For standard diagnostic radiographic voltages, every 0.1 mm copper equals about 3mm aluminium [3]. Reduction in patient ESAK of over 50% has been achieved without any loss in image quality with the installation of additional filtration of 3 mm aluminium [10]. As a step towards optimisation of radiographic practice it would be useful if follow up studies are conducted to evaluate reductions in ESAK following the use of additional filtration.

This study included patients of varying ages, though of different body sizes. To compare patient studies, results need to be presented in a comparative way. It has been suggested that equating ESAK with equivalent patient diameter (EPD) provided a means of comparing patient doses, which takes into account the different body sizes, irrespective of age [11]. The author’s own experience has shown that most radiographers do not favour calculating EPDs manually before each x-ray examination. Dividing the children into age groups is not ideal but it is easy and practical to be employed by radiographers [3,12]. However, with current development in x-ray equipment which incorporates automatic exposure charts based on radiographic technique, patient’s weight and height, exposure factors appropriate for the x-ray examination can be automatically selected. Interestingly, the results showed that older children with larger body thicknesses received lesser doses than younger children. It is common for radiographers to use higher kVps with reduced mA for older children, which substantially reduced the patient dose.

The ESAKs recorded in this study following changes in tube potential were comparable with other studies [10,12,13]. A survey of ESAKs in adult chest radiography in Malaysia found wide variation in doses [2]. The mean dose for an average adult was around 0.28 mGy. The range of doses recorded in this survey was close to these values. Paediatric radiology cannot be compared with adult radiography due to the differences in anatomy, selection of exposure factors and co-operativeness of patients during radiographic examinations. Nonetheless if the paediatric doses are similar to adult limits, this indicates that unsuitable equipment or radiographic techniques are being used, indicative of unacceptable radiographic practice. This study concentrated on the change in tube potential while other methods of dose reduction and better optimisation of radiographic technique should be reviewed to bring the doses to recommended levels [14].

Evaluation of image quality based on acceptable visualisation of anatomical features provided a satisfactory way of evaluating diagnostic acceptability. No marked differences in image quality were found when assessed by using image quality criteria. The general finding in the survey indicated that with the use of higher kVps, images of acceptable subjective value can be obtained with substantial decrease in ESAKs.

However analysis of the specific criteria indicated variations in the visualisation of the anatomical structures. In the second phase of the study, wider variations in scores were noted in the visualisation of the trachea and proximal bronchi (Criterion B). This finding was discussed with both the assessors and both agreed that the poor visualisation of these structures was due to lack of film density. They also found the grading scale quite confusing for this item especially if only one of the two proximal bronchi was seen or if the trachea was visible without the clear visualisation of the bronchi. Similarly there was overall reduced visualisation of the mediastinal structures due to inadequate density. Dark images are usually accompanied by the lack of reproduction of the peripheral vessels while light images mainly affect the visualisation of trachea, proximal bronchi, retrocardiac lung and mediastinum, parameters which are dependent on the optimum tube potential and mAs [15]. As this study was confined to increase in kVps with reductions in mAs, it is possible that some patients would have required an increase in mAs values to provide adequate film densities and better visualisation of these structures.

Densitometric measurements did not show significant differences between both techniques. Although it would have been expected that there would have been reduced radiographic contrast during Technique B, the mean density differences between the 3 areas of density measurements showed no differences. Taking readings from the 3 areas of the chest posed a problem especially in some images where the width of the intercostal space was too small to obtain an accurate reading with the available densitometer. However this study did indicate that images of acceptable radiographic densities can achieved with increases in kVp and reduction in mAs.

Subjective evaluation of the images was performed to identify specific anatomical features. However it would be useful to study whether the good visualisation of anatomical structures correlates with the clinical efficacy of the images and whether it improves the confidence of radiologists and clinicians to reach a confirmative diagnosis.

## CONCLUSION

This study has demonstrated a wide variation in patient doses and radiographic technique during paediatric chest radiography. Significant dose reduction and improvement in image quality was achieved through appropriate increase in tube potential. It is recommended that the kVp range should follow the recommended values of between 60 and 80 kVp for children within the 1 to 15 years age range. Assessment of radiographic images can be evaluated through comparisons with the recommended image criteria providing a means of continuous monitoring of image quality in the radiology department.

Variations in ESAK found in this study are unacceptable and call for better optimisation of radiographic technique. Responses from the various departments in the country indicated that the current radiographic technique in children is far from optimal and highlights the potential for substantial dose reductions. Further studies will be undertaken to compare inter-departmental variations and identify centres which require a change in their existing radiographic practice. Interestingly, the present survey has helped to influence the formulation of new exposure factors consistent with recommended values of tube potential for paediatric patients in the department where the study was done.

This study, being the first of its kind in Malaysia, will serve to educate radiographers and radiologists so that patient doses can be reduced substantially and while simultaneously maintaining image quality with no additional cost implication. The findings of this study will be highlighted to those involved in medical imaging and radiography of children so as to formulate and ensure the effective implementation of regulations defining acceptable standards of good radiographic practice during paediatric radiography.
